# The median effective dose of propofol combined with butorphanol during artificial abortion: a randomized controlled trial

**DOI:** 10.3389/fmed.2023.1226495

**Published:** 2023-11-27

**Authors:** Yuling Zheng, Jinping Huang, Ying Mai, Xiaoling Li, Zhongqi Zhang

**Affiliations:** ^1^Department of Anesthesiology, The Affiliated Shunde Hospital of Jinan University, Foshan, China; ^2^Department of Anesthesiology, The Seventh Affiliated Hospital of Southern Medical University, Foshan, China

**Keywords:** propofol, butorphanol, median effective dose, painless abortion, up-and-down method

## Abstract

**Objective:**

Propofol-opioids are the most common drug combination and can reduce the dose of propofol and the incidence of adverse events in painless artificial abortion. We hypothesized that butorphanol may reduce the median effective dose (ED_50_) of propofol, propofol injection pain, and postoperative uterine contraction pain.

**Methods:**

This was a randomized, double-blind, controlled study. A total of 54 female patients, who had ASA I or II, aged 18–49 years, undergoing painless artificial abortion, were randomly assigned into two groups, namely, Group P (propofol) and Group PB (propofol plus 10 μg/kg butorphanol). According to the pre-experiment, the initial dose of propofol for the P and PB groups was 3 and 2.5 mg/kg, respectively, with a dose gradient of 0.25 mg/kg. The ED_50_ of propofol was analyzed using probit regression analysis. The total propofol dose consumed, recovery time, and anesthesia-related adverse events were also recorded.

**Results:**

There were 25 and 29 patients in the P and PB groups, respectively. The ED_50_ (95% CI) of propofol for artificial abortion were 2.477 (2.186–2.737) and 1.555 (1.173–1.846) mg/kg in the P and PB groups, respectively. The total propofol dose consumed was (150.7 ± 21.7) mg and (110.4 ± 28.2) mg in the P and PB groups, respectively (*P* < 0.001). Compared with the P group, injection-site pain (76 vs. 20.7%) and uterine contraction pain (72 vs. 6.9%) in the PB group had a significant decrease (*P* < 0.001).

**Conclusion:**

Combination of propofol with 10 μg/kg butorphanol reduced the ED_50_ of propofol and decreased the incidence of propofol injection-site pain and postoperative uterine contraction pain during painless artificial abortion compared with propofol alone.

**Clinical trial registration:**

https://www.chictr.org.cn/showproj.html?proj=166610, identifier: ChiCTR2200059795.

## 1 Introduction

Artificial abortion is a relatively fast procedure and can be completed within 3 to 5 min. However, cervical dilation and uterine suction cause intense pain, and some patients exhibit involuntary limb movements, which may increase the risk of uterine perforation (1). Therefore, artificial abortion frequently requires general anesthesia to eliminate the patient's physical discomfort during the procedure.

Propofol, a sedative-hypnotic drug with a rapid onset of action, has been widely used in outpatient surgery or examination anesthesia. However, propofol can cause adverse reactions such as respiratory and circulatory depression, increasing the risk of side effects in high-risk patients (2, 3). When compared to propofol alone, propofol combined with low-dose opioids can provide effective analgesia while lowering the propofol dose (4, 5). Therefore, propofol-opioid combinations can minimize the adverse reactions of high-dose propofol. Butorphanol, a synthetic opioid, exerts analgesic and sedative effects via kappa receptor agonist activity. The most common related adverse reactions to butorphanol include nausea, vomiting, and dizziness, which are also dose-dependent (6, 7). Butorphanol has recently been widely used in outpatient surgery due to its advantages of sedative and analgesic effects with a lower degree of respiratory depression (8, 9).

Sedative and analgesic drugs are the most common drug combinations for painless artificial abortions. However, light or deep anesthesia may cause severe adverse events. Thus, it is necessary to discuss the optimal dose of propofol. We will apply propofol/propofol combined with 10 μg/kg butorphanol in painless artificial abortion to assess the median effective dose (ED_50_) of the propofol in inhibiting cervical dilatation. It is expected to provide a reference for the safety and rational use of the drug in painless artificial abortions without relevant research.

## 2 Materials and methods

### 2.1 Study design and patients

This was a randomized, double-blind, controlled study. The study was approved by the Medical Ethics Committee of the Affiliated Shunde Hospital of Jinan University (number: JDSY-LL-2022005, dated 10 April 2022) and was also registered at www.chictr.org.cn (number: ChiCTR2200059795, dated 11 May 2022; date of the first patient enrollment, 12 May 2022).

### 2.2 Inclusion and exclusion criteria

The inclusion criteria for this study are as follows: elective artificial abortion; American Society of Anesthesiologists (ASA) class I or II; clinically confirmed early pregnancy by color Doppler ultrasound (<12 weeks); age between 18 and 49 years; and body mass index (BMI) between 18 and 30 kg/m^2^.

The exclusion criteria for this study include refusal to participate; ASA class III or higher; allergy to drug-related substances; severe liver, kidney, cardiopulmonary, or central nervous system dysfunction; a procedure time exceeding 10 min; and long-term use of sedative or analgesic medications.

### 2.3 Anesthesia management and surgical procedure

Enrolled patients were randomly assigned into one of the two groups: Group P (propofol) and Group PB (propofol plus 10 μg/kg butorphanol). Patients in group P and group PB received intravenous propofol (Nhwa Pharma Corporation, 20 ml: 0.2 g, lot number: BB220308) for sedation. Patients in group PB received intravenous 10 μg/kg of butorphanol (Jiangsu Hengrui Medicine Co., China, 1 ml:1 mg, diluted to 10 ml with normal saline, lot number: 220129BP) at least 5 min before intravenous propofol; patients in group P received intravenous an equal volume of normal saline. The maximal consumption of butorphanol was 1 mg.

Patients fasted for more than 8 h, and drinking was forbidden for at least 2 h. After entering the operating room, venous access was obtained at the dorsum of the left hand using an intravenous infusion needle with a diameter of 0.6 mm. The oxygen was administered at a flow rate of 3–5 L/min via a nasal straw. The electrocardiogram (ECG), non-invasive blood pressure (NIBP), and peripheral capillary oxygen saturation (SpO_2_) were measured.

According to reports, cervical dilation was the most painful part of the procedure (10). Cervical dilatation with a cervical dilating rod was defined as “Ineffective” if the patient has body movement and it affects the gynecologist's operation. Therefore, the propofol dosage was increased for the next patient. Otherwise, it was defined as “Effective,” and the propofol dosage was decreased for the next patient. According to the pre-experiment, the initial dose of propofol for the P and PB groups was 3 and 2.5 mg/kg, respectively, with a dose gradient of 0.25 mg/kg. After an “Effective” sedation, propofol dosage was decreased by 0.25 mg/kg for the next patient. However, if the sedation was “Ineffective,” the propofol dose was increased by 0.25 mg/kg for the next patient.

All patients were transferred to the post-anesthesia care unit (PACU) following the procedure until their consciousness was regained. Continuous monitoring of ECG, NIBP, and SpO_2_ was performed at 5-min intervals for a minimum duration of 30 min. The criteria for transfer out of the PACU encompassed stable vital signs, independent ambulation, and the absence of evident adverse reactions.

### 2.4 Outcome assessments

The primary outcome measure was ED_50_ of the propofol.

The secondary scales were mean arterial pressure (MAP), heart rate (HR), and SpO_2_ after entering the operating room (T1) and after intravenous administration of propofol (T2). Initial propofol dosage, total propofol dosage, procedure duration, and recovery time were recorded. Adverse events included respiratory depression (SpO_2_ < 90%), hypotension, bradycardia, injection-site pain, uterine contraction pain, postoperative nausea and vomiting (PONV), and dizziness.

Injection-site pain was defined as pain in the backhand or ipsilateral arm escape reflex. The recovery time was between the last propofol injection and the eye-opening on command.

The adverse events were handled as follows: hypotension, defined as a 20% reduction in MAP compared to baseline or <60 mmHg, was treated with intravenous ephedrine at a dosage of 6–12 mg. Bradycardia, indicated by an HR lower than 50 beats/min, was addressed with intravenous atropine at a dosage of 0.25–1 mg. Respiratory depression, characterized by SpO_2_ below 90%, was managed by maintaining ventilation with either a mask or a laryngeal mask. PONV was treated with intravenous tropisetron at a dosage of 2 mg. Uterine contraction pain, assessed using the visual analog scale (VAS) ranging from 0 (painless) to 10 (severe pain), score ≥ 4 intravenous sufentanil at a dosage of 3–5 μg.

### 2.5 Blinding method

The assignments for randomization were generated by computers, and subsequently, group information was concealed within an opaque envelope. The same experienced gynecologist and anesthesiologist performed all surgical procedures and anesthesia. The butorphanol was diluted with normal saline to a volume of 10 ml, which appeared colorless and odorless. The 10-ml transparent syringe without any label (butorphanol or normal saline) was placed in a tray together with propofol for the recruited patient. The distribution of drugs was the responsibility of an independent researcher.

### 2.6 Statistical analysis

Patients' sample sizes were calculated using Dixon's up-down method (11). The approach required at least seven crossovers (Effective to Ineffective) for statistical analysis.

All statistical analyses were performed using SPSS version 20.0 (Inc., Chicago, IL, United States). The data were presented as means ± standard deviations (SD), median [interquartile ranges, IQR], or many patients (*n*), depending on the distribution of the data. Normally distributed continuous variables were compared using the Student's *t*-test, while the Mann-Whitney U test was used for non-normally distributed continuous variables. Categorical variables were compared using the chi-square or Fisher's exact probability test. The ED_50_ of propofol and its 95% confidence interval (CI) were analyzed using probit regression analysis. *P* < 0.05 indicated a statistically significant difference.

## 3 Results

### 3.1 Patient characteristics

**Figure 1:**
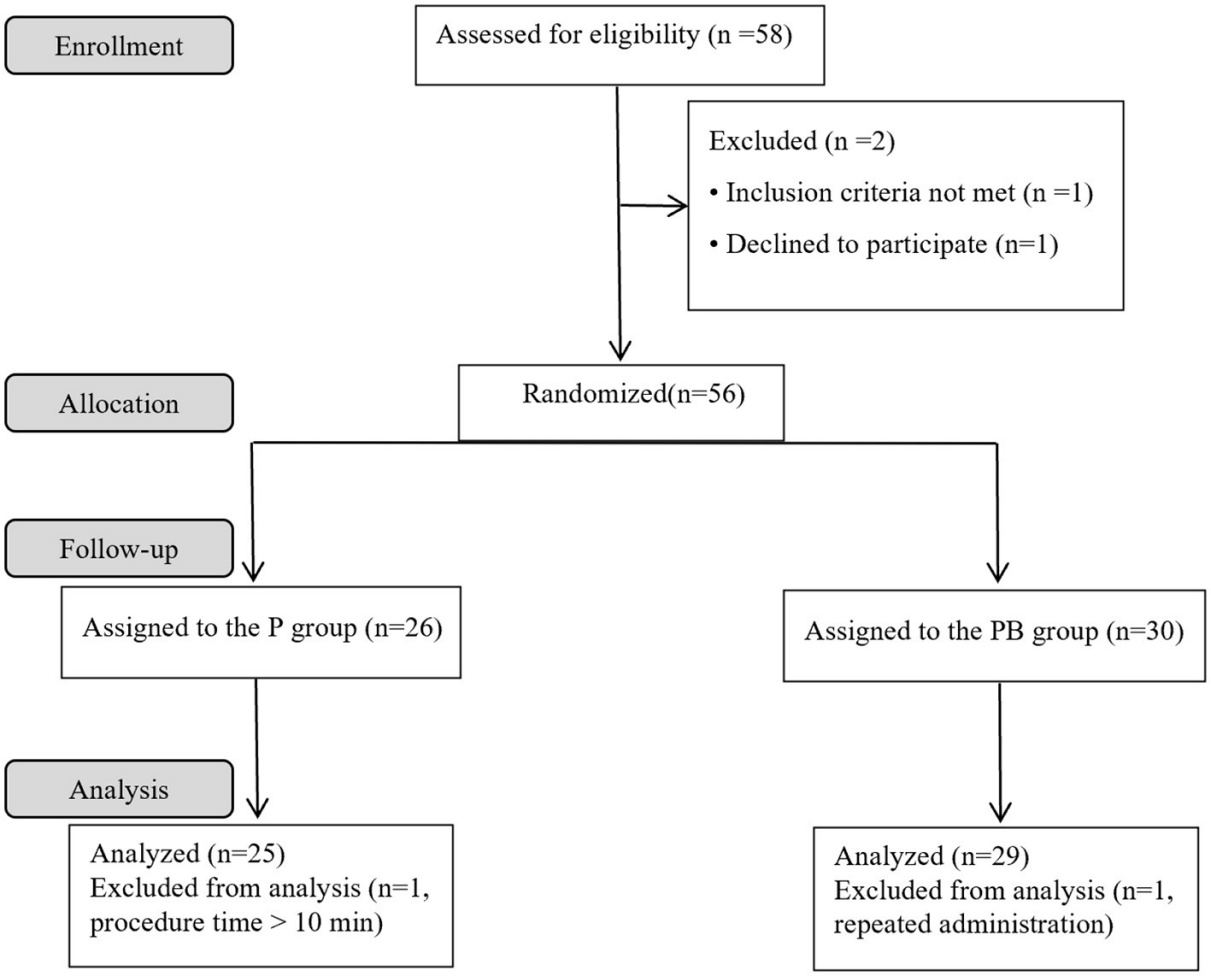
Study flowchart.

A total of 58 female patients were enrolled in the present study. Four patients were excluded, and 54 patients completed the study successfully. Figure 1 depicts the study flowchart. Table 1 demonstrates patients' characteristic data for all patients. There were no statistically significant differences (*P* > 0.05) between the two groups in terms of ASA, age, height, weight, BMI, gestational day, number of times pregnant, number of cesarean sections, number of vaginal deliveries, and number of abortions.

**Table 1:** Patients' characteristics.

**Characteristic**	**Group P, *n* = 25**	**Group PB, *n* = 29**	***P*-value**
ASA (I/II)	24/1	27/2	1.000-
Age (years)	29.9 ± 6.8	32.1 ± 6.7	0.242
Height (cm)	157.5 ± 4.4	155.7 ± 4.9	0.176
Weight (kg)	52.7 ± 7.1	51.4 ± 6.0	0.481
BMI (kg/m^2^)	21.2 ± 2.3	21.1 ± 2.3	0.992
Gestational day	37.9 ± 3.0	36.4 ± 2.8	0.598
Number of times pregnant	3 [2–4]	4 [2.5–4]	0.668
Number of cesarean sections	1 [1–2]	1 [0–2]	0.920
Number of vaginal deliveries	0 [0–0.5]	0 [0–0]	0.711
Number of abortions	2 [1–2]	2 [1–2]	0.861

Data are expressed as means ± standard deviations (SD) or median [range]. Student's t-test or Mann-Whitney U test was used to assess differences.

### 3.2 ED_50_ of propofol

**Figure 2:**
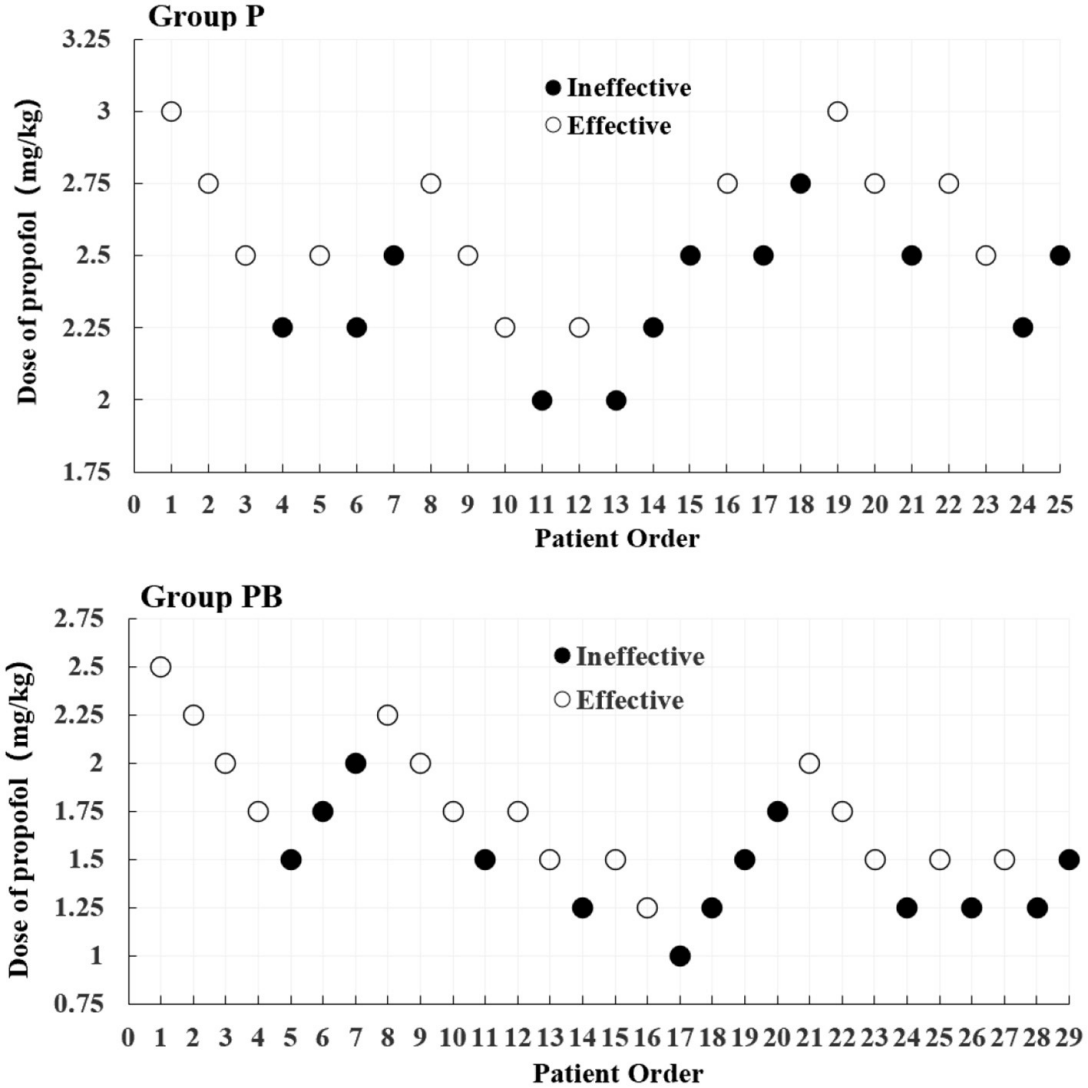
Dixon's up-down method plots for two groups. The white and black dots represent the “Effective” and “Ineffective” patient orders, respectively.

After seven “Effective/Ineffective” crossovers, the sample size was achieved using the up-and-down method. There were 25 and 29 patients in the P and PB groups, respectively (Figure 2). There were 12 and 13 patients who were ineffective and given propofol as rescue therapy in the P and PB groups, respectively. The ED_50_ (95% CI) of propofol for artificial abortion were 2.477 (2.186–2.737) and 1.555 (1.173–1.846) mg/kg in the P and PB groups, respectively (Table 2). Compared with the P group, the ED_50_ of the propofol in the PB group decreased by 37.2%.

**Table 2:** ED_50_ of propofol and their respective 95% CI between the two groups.

**Group**	**Group P**	**Group PB**
ED_50_ (mg/kg)	2.477	1.555
95% CI (mg/kg)	2.186, 2.737	1.173, 1.846

The probit regression analysis was used to determine the ED_50_ of propofol and their respective 95% CI.

### 3.3 Perioperative outcomes

Table 3 displays the perioperative outcomes. The initial and total dosage of propofol consumed in the P group was significantly higher than that in the PB group (131.5 ± 22.2 vs. 84.9 ± 23.6 mg, 150.7 ± 21.7 vs. 110.4 ± 28.2 mg, *P* = 0.000, respectively). The procedure duration (3.9 ± 1.1 vs. 3.6 ± 0.9 min, *P* = 0.237) and recovery time (5.6 ± 1.8 vs. 6.8 ± 1.7 min, *P* = 0.176) were not significantly different between the two groups.

**Table 3:** Comparison of perioperative outcomes between the two groups.

**Items**	**Group P**	**Group PB**	***P*-value**
Initial dose of propofol (mg)	131.5 ± 22.2	84.9 ± 23.6	0.000
Total dose of propofol (mg)	150.7 ± 21.7	110.4 ± 28.2	0.000
Duration of procedure (min)	3.9 ± 1.1	3.6 ± 0.9	0.237
Recovery time (min)	5.6 ± 1.8	6.8 ± 1.7	0.176

Data are expressed as mean ± SD. The total dose of propofol is the initial dose plus the additional dose. Student's t-test was used to assess differences.

### 3.4 Hemodynamic changes at two different time points

HR, MAP, and SpO_2_ levels at two different time points decreased to a certain extent (*P* < 0.05) after intravenous propofol in the two groups. In the P group, the MAP experienced an average drop of 15.7%, whereas the PB group achieved only a 12% drop (Table 4).

**Table 4:** Comparison of HR, MAP, and SpO_2_ of the two groups at different time points.

**Parameters**	**Group**	**Time point**		***P*-value**
		**T1**	**T2**	
MAP (mmHg)	Group P	86.1 ± 12.4	72.5 ± 11.1	0.000
	Group PB	87.9 ± 12.6	77.3 ± 9.2	0.023
HR (beats/min)	Group P	80.2 ± 11.4	79.8 ± 11.3	0.911
	Group PB	84.7 ± 14.7	76.9 ± 9.4	0.019
SpO_2_ (%)	Group P	99.5 ± 0.7	97.9 ± 3.0	0.011
	Group PB	99.5 ± 0.7	98.3 ± 2.3	0.009

Data are expressed as mean ± SD. T1: 5 min after entering the operating room; T2: immediately after IV injection of propofol. Student's t-test was used to assess differences.

### 3.5 Anesthesia-related adverse events

There were no statistically significant differences (*P* > 0.05) between the two groups in anesthesia-related adverse events of SpO_2_ < 90% (8 vs. 3.4%), hypotension (28 vs. 17.2%), bradycardia (4 vs. 0%), dizziness (4 vs. 10.3%), and PONV (4 vs. 7.9%). Compared with the P group, we discovered a significant decrease in both injection-site pain (76 vs. 20.7%) and uterine contraction pain (72 vs. 6.9%) in the PB group; however, this pain was mostly mild (Table 5).

**Table 5:** Anesthesia-related adverse events.

**Adverse events**	**Group P, *n* = 25**	**Group PB, *n* = 29**	***P*-value**
SpO_2_ < 90%	2 (8)	1 (3.4)	0.591
Hypotension	7 (28)	5 (17.2)	0.343
Bradycardia	1 (4)	0 (0)	0.463-
Injection-site pain	19 (76)	6 (20.7)	0.000
Mild pain	18	6	
Moderate pain	1	0	
Uterine contraction pain	18 (72)	2 (6.9)	0.000
Mild pain	12	2	
Moderate pain	6	0	
Dizziness	1 (4)	3 (10.3)	0.615
PONV	1 (4)	2 (7.9)	1.000

Data are expressed as n (%) or median [range]. A chi-square or Fisher's exact probability test was used to assess differences.

## 4 Discussion

Among the many methods for determining ED_50_, Dixon's up-and-down method is rapid and easy and can draw solid conclusions with a relatively small sample size (11). The minimum effective dose can achieve the appropriate depth of anesthesia while reducing drug dosage and the incidence of adverse events. In our study, the trial was terminated when the 25th and 29th female patients reached seven crossover points in the two groups, respectively. The results depict that the ED_50_ of propofol was 2.477 and 1.555 mg/kg, respectively, using probit regression analysis.

All patients were operated on by an experienced gynecologist with ten years of experience to avoid surgical skills affecting the research results. The operation time was controlled within 10 min. When conducting an up-and-down clinical trial, it is important to provide rigorous safeguards. Otherwise, insufficient drug administration may result in serious adverse events if some patients are particularly sensitive to pain stimulation. Propofol, combined with opioids, is the most common drug for intravenous anesthesia in outpatient gynecological procedures. Combined with opioids, they can reduce sedative-hypnotic drug requirements during surgical abortion (12). The analgesic effect of butorphanol is approximately 3.5–7 times that of morphine (13). It has a stronger analgesic effect on women than on men (14), especially suitable for visceral pain (15). When compared with other opioids such as fentanyl or sufentanil, butorphanol presents specific advantages in surgical abortion. Butorphanol, like other opioids, causes adverse reactions such as respiratory depression, postoperative nausea and vomiting, awakening delay, dizziness, etc. Therefore, the selection of an appropriate dosage is particularly important. A dose of butorphanol at 9.07 μg/kg was deemed appropriate for sedating during gastrointestinal endoscopy procedures (9). Accordingly, 10 μg/kg butorphanol was chosen for this study. Some studies show that when propofol was combined with 0.2 μg/kg sufentanil or 0.2 mg/kg nalbuphine in hysteroscopy, the ED_50_ of propofol was 1.651 and 1.658 mg/kg, respectively (16, 17). These findings are consistent with our results. Our results showed that the ED_50_ of propofol in group PB decreased by 37.2% (2.477 vs. 1.555 mg/kg) compared with group P. Although propofol combined with opioids is a preferred option during procedural sedation, taking into account the differences of individuals across geographic regions and clinical settings, propofol anesthesia alone is still a superior alternative, and the findings of this study can serve as valuable references for painless artificial abortion.

Propofol, an intravenous sedative-hypnotic with rapid onset, deep sedative efficacy, and rapid recovery time, has been widely used in outpatient surgery, including in children and the elderly. Painless artificial abortion is one of the most common outpatient surgeries under procedural sedation, and most patients receive propofol. However, its clinical application is limited by injection-site pain, respiratory depression, and hemodynamic instability (18–20). Our research results also exhibited that HR, MAP, and SpO_2_ declined after intravenous propofol administration. The latest research reveals that the incidence of propofol-induced injection pain was 66.3% (21). The most effective intervention to relieve propofol injection pain was pretreatment with opioids, lidocaine, or 5-HT3 receptor antagonists (22–24), which can increase comfort during anesthesia and improve patient satisfaction. Uterine contraction pain is a common complication after abortion surgery (1), and opioids are one of the most effective methods to reduce postoperative uterine contractions (25, 26). Our research discovered that propofol-induced injection pain decreased from 76 to 20.7% and postoperative uterine contraction pain decreased from 72 to 6.9% after pre-treatment with butorphanol. We can observe that pretreatment with 10 μg/kg butorphanol for at least 5 min can significantly reduce the incidence of propofol-induced injection pain and postoperative uterine contraction pain.

Hypotension is one of the most common adverse reactions during propofol sedation. If propofol is used alone, 35% of patients experience one or more hypotensions during colonoscopy (3). Moreover, studies have demonstrated that 55% of patients who received 3 μg/ml of propofol via target-controlled infusion (TCI) experienced hypotension (27). Our results revealed that the incidence of hypotension in both groups was 28 and 17.2%, respectively. It can be seen from the result that the incidence of hypotension can be reduced to some extent by butorphanol pretreatment; this may be related to the reduced usage of propofol. There is no need to treat with vasoactive medication, and the patient can recover relatively quickly. The average recovery time was approximately 6 min (5.6 vs. 6.8 min, *p* > 0.05), and there was also no statistically significant difference in the incidence of dizziness (4 vs. 10.3%) or PONV (4 vs. 7.9%) after waking up within 30 min but not receiving any medication between the two groups. These results demonstrate that propofol combined with 10 μg/kg butorphanol is effective and safe for painless artificial abortion.

The present study has several limitations. First, the patient's age, pregnancy history, production history, and other factors may affect propofol dosage and adverse reactions. Further research is necessary to determine if there will be differences in the effective dose of propofol that inhibits cervical dilation. Second, the effect of other doses of butorphanol on the effective dose of propofol and the incidence of adverse reactions remain unexplored. Third, due to the combination of propofol and opioids is the most frequently used drug combination in procedural sedation, the applicability of the ED_50_ of propofol (propofol used alone) in clinical practice may be limited.

## 5 Conclusion

The combination of propofol with 10 μg/kg butorphanol reduced the ED_50_ of propofol and decreased the incidence of propofol injection-site pain and postoperative uterine contraction pain during painless artificial abortion compared with propofol alone.

## Data availability statement

The original contributions presented in the study are included in the article/supplementary material, further inquiries can be directed to the corresponding author.

## Ethics statement

The studies involving humans were approved by the Medical Ethics Committee of the Affiliated Shunde Hospital of Jinan University. The studies were conducted in accordance with the local legislation and institutional requirements. The participants provided their written informed consent to participate in this study.

## Author contributions

ZZ conceived and designed the study. YZ wrote the manuscript. YM contributed to data collection. JH performed the data analysis and interpretation. XL contributed to the blind design. All authors read and approved the final manuscript.
